# FGF21/sirtuin 1 axis mediates the cardioprotective effects of high‐intensity interval training against doxorubicin‐induced cardiotoxicity by reducing inflammatory and oxidative stress

**DOI:** 10.1113/EP093527

**Published:** 2026-08-04

**Authors:** Mahdiyeh Farahani, Abbas Ali Gaeini, Alireza Ghardashi Afousi, Mahboobeh Borjian Fard

**Affiliations:** ^1^ Department of Exercise Physiology, Faculty of Sport Sciences and Health University of Tehran Tehran Iran

**Keywords:** cardiotoxicity, exercise training, fibroblast growth factor 21, high‐intensity interval training, inflammation, sirtuin 1

## Abstract

Doxorubicin (DOX) is a potent anthracycline chemotherapeutic agent, but its clinical use is limited by cumulative cardiotoxicity. Exercise, particularly high‐intensity interval training (HIIT), has emerged as an effective non‐pharmacological strategy to counteract chemotherapy‐induced cardiac injury. However, the molecular mechanisms underlying the cardioprotective effects of HIIT remain unclear. This study aimed to determine whether HIIT attenuates DOX‐induced cardiotoxicity via activation of the fibroblast growth factor 21 (FGF21)/sirtuin 1 (SIRT1) signalling pathway in rats. Thirty‐three male Wistar rats (200–250 g) were randomly assigned to three groups (*n* = 11 each): control (CONTROL), DOX‐treated sedentary (DOX‐Sed) and DOX‐treated HIIT (DOX‐HIIT). DOX‐induced cardiotoxicity was induced by intraperitoneal DOX injections (12 mg/kg cumulative dose over 12 days). After confirmation of cardiac dysfunction by echocardiography, the DOX‐HIIT group completed an 8 week treadmill HIIT programme (three sessions per week, five intervals of 4 min at 85%–90% of maximal O_2_ uptake separated by 2 min at 50%–60% of maximal O_2_ uptake). Cardiac function, oxidative stress markers, inflammatory cytokines, plasma levels of FGF21 and protein expression of FGFR1 and the SIRT1/AMPK/Nrf2 pathway were assessed. HIIT improved survival (81.8% vs. 63.6% in DOX‐Sed), prevented weight loss and ameliorated left ventricular systolic dysfunction, as evidenced by increased ejection fraction and fractional shortening (*P* < 0.05). HIIT reduced cardiac interleukin‐6, tumor necrosis factor‐α, interleukin‐1β and malondialdehyde levels while elevating antioxidant enzyme activities. Mechanistically, HIIT increased plasma FGF21 and phosphorylated FGFR1 and upregulated SIRT1, LKB1 and phosphorylated AMPK, leading to activation of the Nrf2/HO‐1 antioxidant pathway. Therefore, HIIT‐induced FGF21 has a potential therapeutic effect on cardiac function in DOX‐induced cardiotoxicity through activation of the FGF21/SIRT1/LKB1/AMPK/Nrf2/HO‐1 signalling pathway.

## Abstract (highlights)

- **What is the central question of this study?**
  Does high‐intensity interval training (HIIT) mitigate doxorubicin‐induced cardiotoxicity through activation of the FGF21/SIRT1 signalling axis and its downstream anti‐inflammatory and antioxidant pathways?
- **What is the main finding and its importance?**
  HIIT improved cardiac function and preserved myocardial mass in doxorubicin‐treated rats by reducing inflammatory cytokines and oxidative stress. These protective effects were associated with increased circulating FGF21, activation of FGFR1 and upregulation of the SIRT1/LKB1/AMPK/Nrf2/HO‐1 pathway. These findings identify the FGF21/SIRT1 axis as a key mediator of exercise‐induced cardioprotection and support HIIT as a promising non‐pharmacological strategy to counteract anthracycline‐related cardiac injury.

## 1 INTRODUCTION

Doxorubicin (DOX) is an anthracycline chemotherapeutic agent widely used in the treatment of various malignancies, including breast cancer, lymphomas and sarcomas (Rawat et al., 2021). However, its clinical utility is limited by its dose‐ and time‐dependent cardiotoxicity. This cardiotoxicity may present acutely or develop gradually as left ventricular dysfunction, ultimately leading to heart failure (Schirone et al., 2022). The pathogenesis of DOX‐induced cardiotoxicity (DIC) is multifactorial, primarily involving excessive generation of reactive oxygen species (ROS), mitochondrial dysfunction, impaired ATP synthesis, DNA damage and activation of pro‐inflammatory signalling pathways (Belger et al., 2024). These processes result in cardiomyocyte apoptosis, interstitial fibrosis and long‐term myocardial remodelling. As cancer survival rates continue to rise, strategies to mitigate DOX‐related cardiac injury have become increasingly important to preserve cardiovascular health in cancer survivors (S. Li et al., 2025).

In recent years, exercise has emerged as an effective non‐pharmacological approach to counteract chemotherapy‐induced cardiovascular toxicity (Jin et al., 2022). Among various exercise modalities, high‐intensity interval training (HIIT) has received particular attention for its ability to elicit strong cardiometabolic and cellular adaptations within a relatively short period (Jiménez‐Maldonado et al., 2020). Evidence supports that HIIT enhances endothelial function, promotes mitochondrial biogenesis and upregulates endogenous antioxidant defences. Moreover, HIIT modulates systemic and cardiac inflammation by altering cytokine profiles and suppressing pro‐inflammatory signalling (Bo et al., 2021; Ramez et al., 2019). Collectively, these findings indicate that HIIT might alleviate DOX‐induced cardiac injury; however, the molecular mediators underlying this cardioprotection remain poorly understood.

Fibroblast growth factor 21 (FGF21) has recently emerged as a multifunctional regulator of metabolic homeostasis and cellular stress responses (Luo et al., 2017). Although the liver is a principal source of circulating FGF21, it is also expressed in the heart and skeletal muscle, where it modulates glucose and lipid metabolism, mitochondrial function and oxidative stress resilience (Planavila et al., 2015). Increased FGF21 expression is closely associated with adaptive responses to metabolic and oxidative stress, suggesting a potential cardioprotective role (Tan et al., 2023). Likewise, sirtuin 1 (SIRT1), an NAD^+^‐dependent histone deacetylase, plays a central role in modulating stress resistance, mitochondrial biogenesis and anti‐inflammatory pathways. By deacetylation of key transcriptional regulators, such as peroxisome proliferator‐activated receptor‐ γ coactivator 1α (PGC‐1α), nuclear factor kappa B (NF‐κB) and forkhead box O (FOXO) transcription factors, SIRT1 promotes antioxidant gene expression and suppresses inflammation (Alcendor et al., 2007). FGF21 has been shown to exert cardioprotective effects through activation of mitochondrial SIRT3 signalling, promotion of AMP‐activated protein kinase (AMPK) phosphorylation and induction of FOXO3, thereby reducing mitochondrial dysfunction and ROS accumulation in cardiomyocytes (Jin et al., 2022). Additionally, FGF21 alleviates DIC by enhancing the SIRT1–liver kinase B1 (LKB1) interaction, decreasing LKB1 acetylation and activating AMPK, ultimately upregulating nuclear factor erythroid 2‐related factor 2 (Nrf2)‐mediated antioxidant responses (Wang et al., 2017). These observations highlight FGF21 as a promising therapeutic target for mitigating DOX‐related cardiotoxicity. Emerging evidence further indicates that FGF21 and SIRT1 form a regulatory axis rather than functioning independently; FGF21 can upregulate SIRT1 activity, whereas SIRT1 can potentiate FGF21 signalling, establishing a positive feedback loop that enhances cellular resilience to oxidative and inflammatory stress (Tan et al., 2023). Importantly, exercise intensity appears to be a crucial determinant of the magnitude of FGF21‐ and AMPK‐mediated signalling responses. Higher‐intensity exercise is associated with greater ATP turnover and transient energetic stress, resulting in enhanced activation of AMPK, a central upstream regulator of both SIRT1 activity and FGF21 expression (Hardie, 2015). Clinical evidence indicates that HIIT induces more pronounced activation of AMPK‐dependent pathways and might influence the magnitude and sustained elevations in circulating FGF21 compared with moderate‐intensity continuous exercise (Riahy, 2024a). Animal studies have demonstrated that HIIT improves mitochondrial function and attenuates oxidative stress by increasing cardiac FGF21 receptor expression and activating the SIRT3/AMPK/FOXO3 pathway (Jin et al., 2022). The FGF21/SIRT1 axis is therefore considered a key mediator of exercise‐induced benefits on metabolic tissues and cardiovascular health (Liu et al., 2017). Nonetheless, the specific effects of HIIT on DIC and the FGF21/SIRT1 signalling pathways remain largely unexplored.

The present study was designed to test the hypothesis that the FGF21/SIRT1 signalling axis mediates the cardioprotective effects of HIIT in DIC. Specifically, we aimed to determine whether HIIT mitigates DOX‐induced oxidative stress and inflammation in cardiac tissue through activation of the FGF21/SIRT1 pathway. Elucidating this mechanism might provide new insights into exercise‐based cardioprotective strategies for patients undergoing anthracycline chemotherapy and support the development of integrated interventions to preserve cardiovascular health in this population.

## 2 MATERIALS AND METHODS

### 2.1 Animals and experimental design

Male Wistar rats (200–250 g) were obtained from the Pasteur Institute in Tehran, Iran. Upon arrival, animals were acclimated for 1 week in transparent polycarbonate cages (four rats per cage) in controlled environmental conditions: temperature 20°C–24°C, relative humidity 45%–55%, and a 12 h–12 h light–dark cycle. Rats had ad libitum access to standard chow and water throughout the study. All experimental procedures were approved by the Ethics Committee of the University of Tehran (IR.UT.SPORT.REC.1403.017) and conducted in accordance with the *Guide for the Care and Use of Laboratory Animals* (US National Institutes of Health). Rats were randomly assigned to one of the three groups (*n* = 11 per group): (1) control healthy (CONTROL); (2) doxorubicin‐treated sedentary (DOX‐Sed); or (3) doxorubicin‐treated HIIT (DOX‐HIIT). Cardiotoxicity was induced in DOX‐Sed and DOX‐HIIT groups by intraperitoneal injection of DOX at a cumulative dose of 12 mg/kg (six injections of 2 mg/kg every 48 h over 12 days) (M. Li et al., 2025). Body weight was measured longitudinally throughout the experimental period.

### 2.2 Echocardiography

Two weeks after the first DOX injection, transthoracic echocardiography was performed to confirm cardiotoxicity. Rats were anaesthetized with sodium thiopentone (25 mg/kg, intraperitoneally), and cardiac function was assessed using a Vivid 7 Expert Ultrasound System (General Electric Vingmed Ultrasound, Horten, Norway) equipped with a 10 MHz linear array transducer. Parameters including left ventricular diastolic diameter, left ventricular systolic diameter, end‐diastolic volume (EDV), end‐systolic volume (ESV), ejection fraction (EF) and fractional shortening (FS) were obtained from two‐dimensional and M‐mode, short‐axis images. All echocardiography assessments were performed by an experienced radiologist specializing in small‐animal imaging at the University of Tehran Veterinary Medicine Hospital.

### 2.3 Exercise training programme

Following echocardiographic assessment, maximal oxygen consumption (${\dot{V}}_{O_{2}\max}$) was determined using a graded treadmill test according to established protocols (Ghardashi Afousi et al., 2019; Høydal et al., 2007). The CONTROL and DOX‐Sed groups were placed on the treadmill but did not undergo training. The DOX‐HIIT group performed an 8 week HIIT protocol consisting of three sessions per week. Each 40 min session included a 5 min warm‐up at 40%–50% ${\dot{V}}_{O_{2}\max}$, five intervals of 4 min high‐intensity intervals at 85%–90% ${\dot{V}}_{O_{2}\max}$ interspersed with 2 min of active recovery at 50%–60% ${\dot{V}}_{O_{2}\max}$, and 5 min cool‐down at 40%–50% ${\dot{V}}_{O_{2}\max}$. Training intensity was adjusted weekly by increasing treadmill speed by 0.02 m/s, based on the relationship between running speed and ${\dot{V}}_{O_{2}\max}$ (Ghardashi Afousi et al., 2019).

### 2.4 Euthanasia and tissue collection

Forty‐eight hours after the final training session, rats that survived to the predefined study end point were anaesthetized with thiopentone sodium (25 mg/kg, intraperitoneally). Adequate anesthesia was confirmed by the absence of corneal and pedal withdrawal reflexes. Under deep general anaesthesia, terminal blood samples were collected via percutaneous cardiac puncture using a 21‐ to 23‐gauge needle into serum‐separator tubes. Cardiac tissue samples were obtained consistently from the left ventricular free wall in all animals in standardized conditions. Cardiac tissues were allocated for multiple planned downstream analyses. For molecular analyses, including ELISA and western blotting, a subset of samples (*n* = 5 per group) was randomly selected from the surviving animals within each group to maintain balanced group sizes and comparability across experimental conditions. The remaining cardiac tissues were preserved as paraffin‐embedded samples for potential future histological analyses.

### 2.5 Western blot

Heart tissues were homogenized in Pro‐PRE™ cell lysis buffer (iNtRON Biotechnology, Korea) and centrifuged at 16,000 rpm for 15 min at 4°C. Protein concentrations were determined using the BCA protein assay (iNtRON Biotechnology, Korea). Equal amounts of protein (20 µg) were separated by 10%–12% SDS–PAGE and transferred to polyvinylidene fluoride membranes (162‐0177, Bio‐Rad Laboratories, CA, USA). Membranes were blocked with 5% skim milk (A‐7888, Sigma Aldrich, MO, USA) for 1 h and incubated overnight at 4°C with the following primary antibodies: anti‐SIRT1 (1:1000, ab189494), anti‐AMPK (1:1000, ab32047), anti‐Nrf2 (1:1500, ab313825), anti‐HO1 (1:2000, ab189491), anti‐phospho FGFR1 Y654 (1:5000, ab59194), anti‐β‐actin (1:2500, ab8227, Abcam, Berlin, Germany), anti‐phospho AMPK (A94217, Antibodies), anti‐phospho Ser40‐Nrf2 (1 mg/mL, orb6544, Biorbyt, NC, USA) and anti‐LKB1 (3047S, Cell Signaling Technology, USA). After washing three times with Tris‐buffered saline with 0.1% Tween‐20 (TBST) (15 min each), membranes were incubated with horseradish peroxidase‐conjugated goat anti‐rabbit secondary antibody (1:10 000, ab6721; Abcam) for 2 h at room temperature. Protein bands were visualized using enhanced chemiluminescence (ECL, Thermo Fisher Scientific, USA) and quantified by densitometry using Gel Analyzer software (NIH, USA). Protein expression levels were normalized to β‐actin.

### 2.6 ELISA of inflammatory cytokines

Cardiac tissue concentrations of interleukin‐1β (IL‐1β; R&D Systems, Catalog No. RLB00, Minneapolis, MN 55413, USA), interlukin‐6 (IL‐6; Cusabio, Catalog No. CSB‐E04640r, Houston, TX, USA), and tumor necrosis factor‐α (TNF‐α; R&D Systems, Catalog No. RTA00, Minneapolis, MN 55413, USA) were determined using commercial ELISA kits following the manufacturers’ instructions.

### 2.7 Oxidative stress measurements

Lipid peroxidation was assessed using the thiobarbituric acid reactive substances (TBARS) assay, which quantifies malondialdehyde (MDA)–TBA adducts spectrophotometrically (R&D Systems, Bio‐Techne, Catalog No. KGE013, Minneapolis, MN 55413, USA). Results were normalized to protein concentration, and all assays were conducted in duplicate. Antioxidant enzyme activities were assessed spectrophotometrically using commercial kits for superoxide dismutase (SOD; Catalog No. MBS036924, MyBioSource, San Diego, CA, USA), catalase (CAT; Catalog No. E‐BC‐K301‐S, Elabscience, Houston, TX, USA), reduced glutathione (GSH; Catalog No. E‐EL‐0026, Elabscience, Houston, TX, USA) and oxidized glutathione (GSSG; Catalog No. E‐BC‐K097‐M, Elabscience, Houston, TX, USA), following the manufacturer's protocols.

### 2.8 ELISA of FGF21

Plasma levels of FGF21 were measured using commercial ELISA kits (R&D Systems, Catalog No. MF2100, Minneapolis, MN 55413, USA) according to the manufacturer's instructions.

### 2.9 Statistical analysis

All data are presented as the mean ± SD. Statistical analyses were performed using SPSS v.25.0 (IBM Corp., USA) and GraphPad Prism v.9 (GraphPad Software, USA). Data normality was assessed using the Shapiro–Wilk test. Differences among groups were evaluated using one‐way ANOVA followed by Tukey's *post hoc* test. A value of *P* < 0.05 was considered statistically significant.

## 3 RESULTS

### 3.1 HIIT ameliorates body weight loss, cardiac dysfunction and loss of myocardial mass in DIC

Survival rates were analysed using Kaplan–Meier survival curves and a log‐rank test (Figure 1a). During the 8 week intervention, mortality occurred in the DOX‐treated groups. Specifically, four animals died in the DOX‐Sed group, resulting in a survival rate of 63.6%, and two animals died in the DOX‐HIIT group, resulting in a survival rate of 81.8%. Therefore, final group sizes at the predefined study end point were *n* = 11 (CONTROL), *n* = 7 (DOX‐Sed) and *n* = 9 (DOX‐HIIT). Functional assessments presented in Figure 1 were performed in all surviving animals. Molecular analyses were conducted using a randomly selected subset of surviving animals (*n* = 5 per group), as described in the Materials and Methods section. Rats treated with DOX showed significant body weight loss, which was attenuated in the DOX‐HIIT group (Figure 1b). Although DOX treatment resulted in a significant reduction in body weight compared with the CONTROL group, the mean body weight loss in the DOX‐Sed group was ∼5% throughout the experimental period (from ∼235 to ∼223 g) and remained within the limits of the approved animal welfare protocol. In addition, heart weight, the heart weight‐to‐body weight ratio and the heart weight‐to‐tibia length ratio were significantly reduced in the DOX‐Sed group compared with CONTROL. These parameters were significantly higher in the DOX‐HIIT than in the DOX‐Sed group (Figure 1c,d).

**FIGURE 1:**
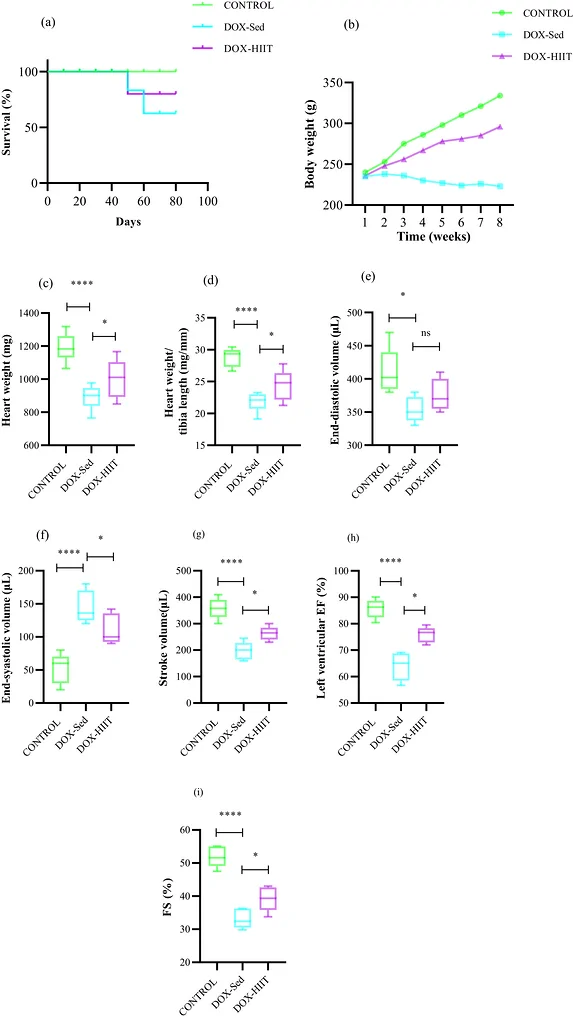
HIIT improves cardiac dysfunction in DIC rats. (a) Body weight changes during the experimental period. (b) Heart weight at study end point. (c) Heart weight‐to‐tibia length ratio. (d–h) Representative echocardiographic images and quantitative analysis of cardiac function parameters. CONTROL *n* = 11, DOX‐Sed *n* = 7 and DOX‐HIIT *n* = 9. *P < 0.05, ****P < 0.0001 and ns, no significance. Abbreviations: CONTROL, control group; DIC, doxorubicine‐induced cardiotoxicity; DOX, doxorubicine; EF, ejection fraction; FS, fractional shorthening, HIIT, high‐intensity interval training; Sed, senentary.

Echocardiographic assessment revealed a significant reduction in EDV in the DOX‐Sed compared with CONTROL, whereas EDV was preserved in the DOX‐HIIT group (Figure 1e). ESV was increased in both the DOX‐Sed and DOX‐HIIT groups compared with CONTROL but was significantly lower in the DOX‐HIIT group than in the DOX‐Sed group (Figure 1f). Stroke volume was reduced in both DOX‐treated groups compared with CONTROL but partly recovered in the DOX‐HIIT group (Figure 1g). Likewise, EF and FS were significantly decreased in both DOX‐treated groups relative to CONTROL; however, both indices were significantly higher in DOX‐HIIT than in DOX‐Sed (Figure 1h,i). Collectively, these findings suggest that HIIT confers cardioprotective effects in DIC rats by improving systolic function and preserving myocardial mass.

### 3.2 HIIT improves cardiac inflammatory signalling through FGF21/SIRT1/AMPK activation

Plasma levels of FGF21 were significantly reduced in both DOX‐treated groups compared with CONTROL (*P *< 0.0001) but were markedly higher in the DOX‐HIIT group relative to DOX‐Sed (*P *< 0.001; Figure 2a). Phosphorylated FGFR1 expression was decreased following DOX administration (*P* = 0.0007) and increased significantly after HIIT (*P* = 0.0108), suggesting that HIIT partly reverse DOX‐induced FGFR1 downregulation (Figure 2b). Likewise, SIRT1 expression was significantly reduced in DOX‐Sed compared with CONTROL (*P* < 0.0001), but increased in the DOX‐HIIT group (*P* = 0.005; Figure 2c). LKB1 expression followed a similar pattern, decreasing after DOX treatment (*P* < 0.0001) and increasing after HIIT (*P* = 0.001; Figure 2d). Total AMPK protein expression did not differ significantly among groups (*P* > 0.05), indicating that DOX and HIIT did not alter overall AMPK abundance. However, phosphorylated AMPK levels were significantly decreased in DOX‐ treated rats compared with the CONTROL group (*P* < 0.001) and were restored by HIIT (*P* = 0.001; Figure 2e). Consistent with these findings, the phospho‐AMPK/total AMPK ratio was significantly reduced in the DOX‐Sed group relative to CONTROL (*P* < 0.0001) and significantly increased in the DOX‐HIIT group compared with the DOX‐Sed group (*P* = 0.003; Figure 2f), indicating enhanced AMPK activation following HIIT.

**FIGURE 2:**
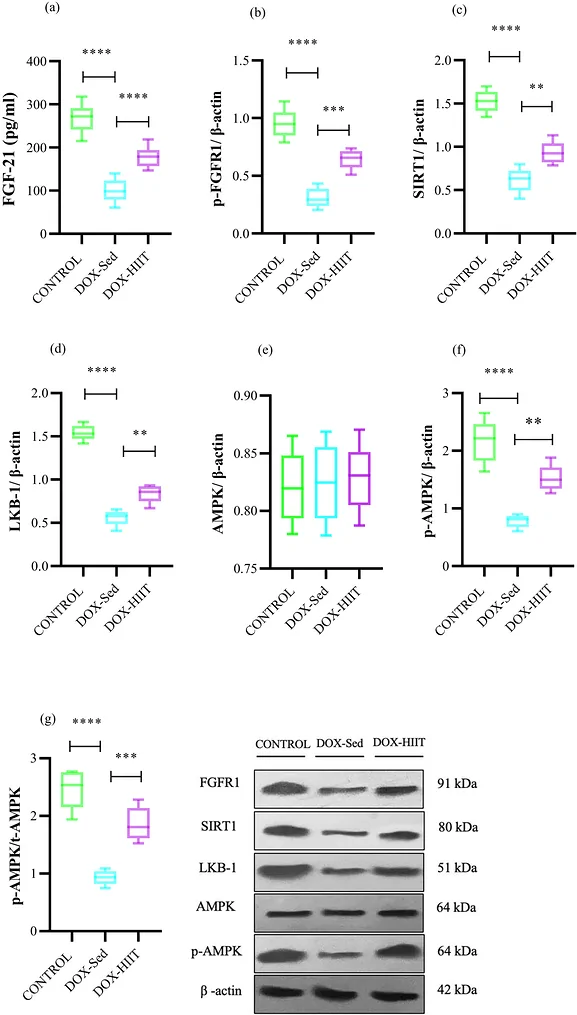
SIRT1 mediated protective of FGF‐21against DIC injury in rats by exercise. (a) Plasma levels of FGF‐21. (b–g) Representative of protein expression of phospho‐FGFR1 (b), SIRT1 (c), LKB (d), total AMPK (e), phospho‐AMPK (f) and the ratio of total AMPK to phospho‐AMPK (g). *n* = 5 in each group. **P < 0.01, ***P < 0.001 and ****P < 0.0001. Abbreviations: AMPK, adnosine monophosphate kinase, CONTROL, control group; DIC, doxorubicine‐induced cardiotoxicity; DOX, doxorubicine; FGF21, fibroblast growth factor 21; HIIT, high‐intensity interval training; LKB1, liver kinase B1; p‐AMPK, phosphorylated adnosine monophosphate kinase; p‐FGFR1, phosphorylated fibroblast growth factor receptor 1; SIRT1, sirtuin 1; shorthening; Sed, senentary.

Regarding inflammatory markers, cardiac IL‐6 levels were significantly elevated in DOX‐Sed compared with CONTROL (*P* < 0.0001) but decreased following HIIT (*P* = 0.042; Figure 3a). Likewise, TNF‐α and IL‐1β concentrations were markedly increased in DOX‐Sed relative to CONTROL (*P* < 0.0001) and significantly reduced after HIIT (*P* < 0.0001; Figure 3b,c). Overall, these results indicate that HIIT elevates plasma FGF21 levels and attenuates cardiac inflammation by activating the SIRT1/LKB1/AMPK signalling pathway.

**FIGURE 3:**
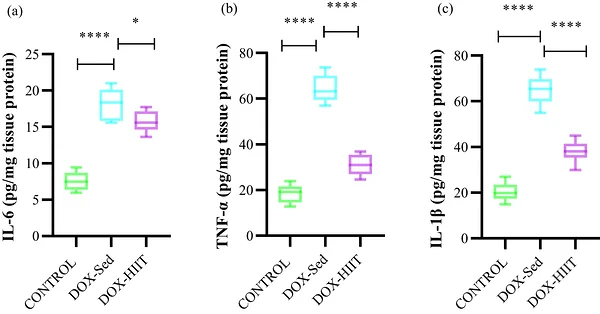
HIIT improves inflammatory cytokines in DIC in rats. Cardiac tissue levels of IL‐6 (a), TNF‐α (b) and IL‐1β (c). *n* = 5 per each group. *P < 0.05 and ****P < 0.0001. Abbreviations: CONTROL, control goup; DIC, doxorubicin‐induced cardiotoxicity; DOX, doxorubicin; HIIT, high‐intensity interval training; IL‐1β, interleukin‐1β; IL‐6, interleukin‐6; Sed, sedentary; TNF‐α, tumor necrosis factor‐α.

### 3.3 HIIT enhances antioxidant defence mechanisms

Biochemical analyses and western blot were performed using cardiac tissue samples obtained from a randomly selected subset of animals (*n* = 5 per group). Cardiac MDA levels were elevated in both DOX‐treated groups compared with CONTROL but were significantly lower in the DOX‐HIIT group than in DOX‐Sed (*P *< 0.0001; Figure 4a). Protein levels of SOD, GSH and GSSG were significantly decreased in both DOX‐treated groups compared with CONTROL; however, all three were elevated in DOX‐HIIT relative to DOX‐Sed after the 8 week intervention (SOD, *P* = 0.001, Figure 4b; GSH, *P* = 0.021, Figure 4d; GSSG, *P* = 0.025, Figure 4e). CAT activity was reduced in both DOX‐treated groups but significantly improved following HIIT (*P* = 0.026, Figure 4c). Total Nrf2 protein expression was unchanged among groups (data not shown), and phosphorylated Nrf2 levels were decreased after DOX exposure (*P* < 0.001) and restored by HIIT (*P* = 0.001; Figure 4f). Consequently, the phospho‐Nrf2/total Nrf2 ratio followed a similar pattern (*P* < 0.0001 for DOX‐Sed vs. CONTROL; *P* = 0.005 for DOX‐HIIT vs. DOX‐Sed; Figure 4g). HO‐1 protein expression was markedly increased in DOX‐treated groups relative to CONTROL (*P* < 0.0001), indicating enhanced oxidative stress. After 8 weeks of HIIT, HO‐1 expression increased further in DOX‐HIIT rats (*P* = 0.023; Figure 4h). Collectively, these findings suggest that HIIT protects against DOX‐induced oxidative damage by upregulating the FGF21/SIRT1/p‐Nrf2/HO‐1 axis and reinforcing the antioxidant defence system in DIC rats.

**FIGURE 4:**
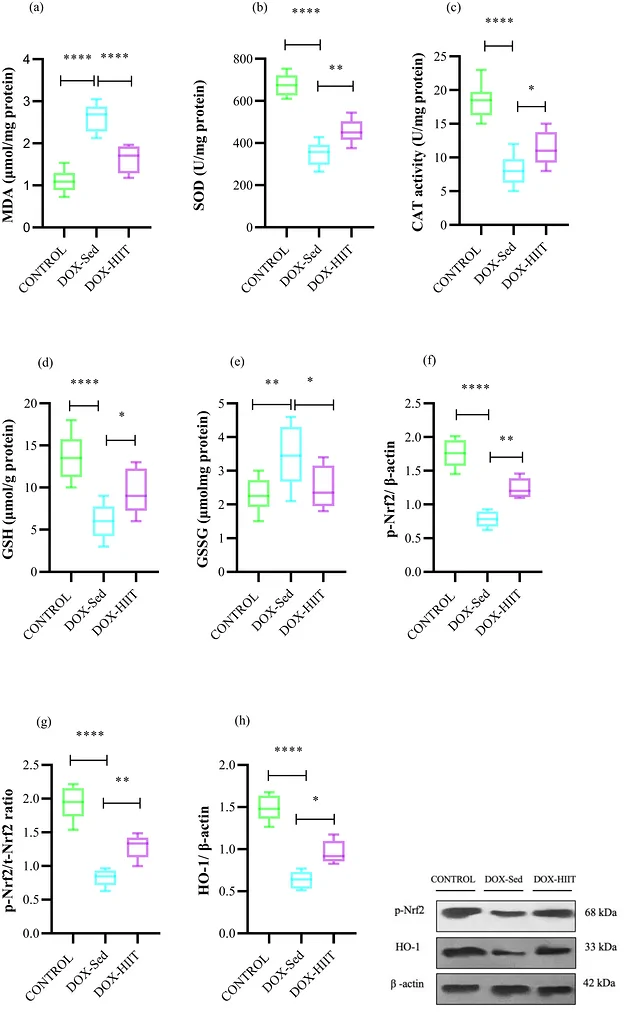
HIIT improves antioxidant system in DIC injury rats via Nrf2 signalling. (a) Cardiac tissue levels of MDA. (b) Cardiac tissue levels of SOD. (c–e) CAT activity of heart tissue (c), GSH (d) and GSSG (e). (f) Protein expression of phospho‐Nrf2. (g) The ratio of total Nrf2 to phospho‐Nrf2. (h) Protein expression of HO‐1. *n* = 5 per each group. *P < 0.05, **P < 0.01 and ****P < 0.0001. Abbreviations: CAT, catalase; CONTROL, control group; DIC, doxorubicine‐induced cardiotoxicity; DOX, doxorubicine; GSH, reduced glutathione; GSSG, oxidized glutathione; HIIT, high‐intensity interval training; HO‐1, heme oxygenase 1; MDA, malondialdehyde, Nrf2, nuclear factor erythroid 2‐related factor 2; p‐Nrf2, phosphorylated nuclear factor erythroid 2‐related factor 2; Sed, sedentary; SOD, superoxide dismutase.

## 4 DISCUSSION

In the present study, we investigated the protective role of HIIT against DIC in rats, with a focus on the metabolic axis of FGF21/SIRT1 and its downstream signalling pathways related to inflammation and oxidative stress. The major findings of our study can be summarized as follows: (1) HIIT attenuated DOX‐induced body weight loss, reduction in myocardial mass and decline in cardiac function as assessed by echocardiography; (2) HIIT was associated with increased plasma FGF21 levels and elevated protein expression of phosphorylated FGFR1, SIRT1, LKB1 and p‐AMPK, alongside reduced pro‐inflammatory cytokines including IL‐6, TNF‐α and IL‐1β; and (3) HIIT was associated with enhancement of the antioxidant defence system, as reflected by lower myocardial MDA levels, increased SOD, GSH, GSSG and CAT activity, restoration of the p‐Nrf2/Nrf2 ratio and upregulation of HO‐1. Taken together, these results suggest that the FGF21/SIRT1 axis might play an important role in mediating the beneficial effects of HIIT in DOX‐induced cardiotoxicity, potentially through modulation of inflammatory and oxidative stress pathways and preservation of myocardial structure and function.

Our data are consistent with and extend prior mechanistic work linking FGF21 and SIRT1 in cardioprotection. Wang et al. (2017) demonstrated that FGF21 treatment in mice attenuated DOX‐induced cardiac dysfunction via the SIRT1/LKB1/AMPK pathway, accompanied by reductions in oxidative stress, inflammation and apoptosis. Likewise, Wang et al. (2022) reported that SIRT1 activation was associated with amelioration of DIC, potentially through targeting SESN2 and enhancing antioxidant responses. To our knowledge, the present study is among the first to examine the effect of HIIT on modulation of the FGF21/SIRT1 axis in the context of DIC, while also incorporating both functional and structural end points (e.g., body weight, cardiac mass and EF/FS) that are consistent with a cardioprotective phenotype. Furthermore, the beneficial effect of exercise in DIC has been supported by meta‐analysis evidence indicating that exercise training is associated with improvements in FS (∼7.4 %) in DOX‐treated rodents (Ghignatti et al., 2021). In the present study, HIIT was associated with improvements in survival, attenuation of body weight loss, preservation of heart weight and heart weight‐to‐body weight/tibia length ratio, in addition to favourable changes in cardiac function indices, including prevention of EDV decline, limitation of ESV elevation, and improvements in stroke volume, EF and FS. These findings suggest that HIIT might contribute to the preservation of both cardiac function and myocardial structural integrity. This interpretation is consistent with previous evidence indicating that exercise preconditioning in DOX models is associated with greater improvements in FS when training is performed before DOX exposure (mean difference ≈ 8.2 %) compared with post‐exposure training (Wang et al., 2020).

One of the most compelling mechanistic insights of our study is the observed activation of the FGF21/SIRT1/LKB1/AMPK signalling axis by HIIT in the DOX‐treated heart. FGF21 has emerged as a metabolic hormone with pleiotropic functions, including anti‐inflammatory and antioxidant properties (Fisher & Maratos‐Flier, 2016; Gimeno & Moller, 2014; Wang et al., 2017). In DOX‐injured myocardium, FGF21 expression is reported to be downregulated, and its restoration has been associated with improvements in cardiomyocyte survival, reduced fibrosis and enhanced myocardial contractility (Wang et al., 2022). In the present study, the DOX‐Sed group exhibited reduced plasma FGF21 levels and a decrease in phosphorylated FGFR1 in cardiac tissue, whereas HIIT was associated with restoration of these markers. This finding suggests that HIIT might act as a non‐pharmacological modulator of FGF21 signalling (Riahy, 2024b). Downstream of FGFR1, SIRT1 upregulation is thought to be an important regulatory step; SIRT1 deacetylates and activates LKB1, which subsequently phosphorylates AMPK, a central regulator of energy metabolism and stress adaptation (Wang et al., 2017, 2022). Our results demonstrated increased SIRT1 and LKB1 expression and an elevated p‐AMPK/AMPK ratio in the DOX‐HIIT group, consistent with activation of this signalling axis. Importantly, total AMPK protein abundance remained unchanged, indicating that HIIT might preferentially enhance AMPK activation rather than its expression level. Furthermore, because cardiac tissues were collected 48 h after the final HIIT session, the observed increase in AMPK phosphorylation is unlikely to represent an acute response to the last exercise bout. Rather, it is likely to reflect a chronic training‐induced adaptation of myocardial energy‐sensing pathways following repeated HIIT exposure throughout the 8 week intervention period. Activation of the Nrf2/HO‐1 antioxidant pathway appears to represent a downstream event of this cascade. The increased phospho‐Nrf2/total Nrf2 ratio and elevated HO‐1 expression observed in the DOX‐HIIT group suggest that HIIT‐induced modulation of FGF21/SIRT1/LKB1/AMPK signalling might converge on canonical antioxidant defence mechanisms. Previous studies have shown that exogenous FGF21 administration is associated with protection against DOX‐induced cardiomyopathy via activation of the SIRT1/LKB1/AMPK pathway, accompanied by reductions in oxidative stress and inflammation (Wang et al., 2017; Xu et al., 2024). Moreover, AMPK activation has been reported to promote Nrf2 nuclear translocation and to upregulate antioxidant gene expression (Xu et al., 2024). Collectively, our findings support a model in which HIIT might stimulate FGF21 production or signalling, leading to the activation of the SIRT1/LKB1/AMPK/Nrf2/HO‐1 pathway. This cascade might contribute to anti‐inflammatory and antioxidant effects, thereby attenuating DOX‐induced oxidative injury and supporting the preservation of cardiac structure and function.

It is well established that DIC is driven by excessive ROS generation, activation of inflammatory pathways (NF‐κB, TNF‐α and IL‐6), mitochondrial dysfunction and subsequent cardiomyocyte death and fibrosis (Yu et al., 2016). In our study, the DOX‐Sed group displayed clear hallmarks of such injury, including elevated IL‐6, TNF‐α and IL‐1β, increased MDA, reduced SOD, GSH and CAT activities, diminished p‐Nrf2, and HO‐1 expression. These findings are consistent with enhanced oxidative stress, inflammatory activation and a compensatory, albeit insufficient, upregulation of cytoprotective pathways. HIIT significantly lowered pro‐inflammatory cytokines, increased antioxidant enzyme activities, reduced MDA and restored Nrf2 signalling. Although the magnitude of recovery differed among oxidative stress markers, both the reduction in MDA and the increase in SOD activity consistently support an overall improvement in myocardial redox balance following HIIT. Given that these biomarkers reflect distinct aspects of oxidative stress and antioxidant defence, complete parallel normalization would not necessarily be expected. Notably, the elevation of HO‐1 expression in the DOX‐HIIT group might reflect an adaptive response to enhanced Nrf2 activity, rather than a simple marker of oxidative injury. This coordinated improvement in redox and inflammatory profiles might plausibly underlie the observed recovery in cardiac mass and function following HIIT. Mechanistically, SIRT1 appears to act as a pivotal upstream regulator linking HIIT to these protective effects. SIRT1 deacetylates NF‐κB, thereby suppressing pro‐inflammatory gene transcription, while also stabilizing Nrf2 and promoting antioxidant gene expression (Ebrahimnezhad et al., 2023; Mercês et al., 2025; Yu et al., 2016). In line with this, increased SIRT1 expression in the DOX‐HIIT group might partly explain the observed cytokine reduction and antioxidant restoration. Moreover, FGF21, a metabolic hormone known for its anti‐inflammatory and antioxidative properties, has been shown to activate SIRT1 and its downstream effectors, LKB1/AMPK/Nrf2 (Wu et al., 2020). Thus, activation of the FGF21/SIRT1/AMPK/Nrf2 axis by HIIT might represent a plausible molecular mechanism through which exercise attenuates DOX‐induced oxidative and inflammatory damage, contributing to improved myocardial integrity and function. Taken together, our findings provide a mechanistic framework suggesting a cardioprotective role of HIIT in DOX‐treated rats. We propose that HIIT might stimulate FGF21 production (possibly from skeletal muscle and/or heart), which might engage FGFR1 signalling; this might lead to SIRT1 activation, deacetylation of LKB1, AMPK phosphorylation, Nrf2/HO‐1 upregulation, and enhancement of antioxidant enzyme systems, all of which might contribute to reductions in inflammatory cytokine expression, ROS accumulation, cardiomyocyte injury and adverse remodelling. This integrated axis might help to explain the preservation of cardiac function and survival benefit observed. From a clinical standpoint, our data suggest that HIIT might represent a promising non‐pharmacological strategy to protect the heart in cancer survivors receiving anthracyclines. Given that conventional cardioprotectants have limited efficacy and potential side‐effects, exercise training, particularly HIIT, might offer a practical and accessible intervention. However, translation to humans will require careful determination of timing (pre‐, peri‐ or post‐chemotherapy), appropriate intensity, safety in immunocompromised patients, and long‐term adherence.

### 4.1 Limitations

Our study has several limitations. First, only male Wistar rats were included; thus, sex specific responses cannot be excluded. Second, although we observed associations between HIIT, FGF21/SIRT1/AMPK signalling and improved outcomes, causal relationships were not tested formally (e.g., by pharmacological or genetic inhibition of SIRT1 or FGFR1). Third, the duration of follow‐up post‐DOX was limited; longer‐term outcomes, such as late‐onset cardiomyopathy or heart failure, were not assessed. Fourth, although we measured numerous end points, we did not quantify fibrotic markers or apoptosis assays directly, which could strengthen mechanistic inferences. In addition, outcomes were assessed over an 8 week period; longer‐term follow‐up is required to determine the durability of cardioprotection and structural remodelling. Furthermore, although rats were studied in controlled experimental conditions, the safety, feasibility and efficacy of HIIT in human cancer patients undergoing DOX therapy remain to be established. An additional limitation is that the present study did not include a non‐DOX HIIT control group. Therefore, the independent physiological effects of HIIT in non‐pathological conditions cannot be separated directly from its modulatory effects in the setting of DIC. However, the primary aim of this investigation was to determine whether HIIT could attenuate DOX‐induced impairments in cardiac FGF21/SIRT1/AMPK/Nrf2 signalling, rather than to characterize baseline exercise adaptations. Accordingly, the experimental design was structured to test differences between DOX‐exposed sedentary and DOX‐exposed trained animals, which directly addresses the pathological interaction of interest. From a statistical perspective, the inclusion of a HIIT‐only group would have enabled additional between‐group comparisons but was not required to evaluate the central hypothesis regarding mitigation of DOX‐induced molecular and functional alterations. Nevertheless, future studies incorporating physiological HIIT control conditions would further clarify the relative contribution of exercise‐specific vs. cardiotoxicity‐specific signalling responses.

## 5 CONCLUSION

In conclusion, this study provides new evidence that HIIT mitigates DIC through activation of the FGF21/SIRT1/LKB1/AMPK/Nrf2/HO‐1 axis, leading to enhanced antioxidant defences and reduced inflammation. These findings position the FGF21/SIRT1 axis as a crucial mediator of exercise‐induced cardioprotection and support the development of HIIT‐based interventions for anthracycline‐related cardiac injury.

## AUTHOR CONTRIBUTIONS

Study conception: Alireza Ghardashi Afousi and Abbasali Gaeini. Design of the research: Alireza Ghardashi Afousi and Abbasali Gaeini. Performed experiments: Mahdieh Farahani. Data analysis: Mahdieh Farahani. Data interpretation: Mahboobeh Borjian Fard and Alireza Ghardashi Afousi. Drafted manuscript: Alireza Ghardashi Afousi. Prepared figures and tables: Mahboobeh Borjian Fard. All authors have read and approved the final version of the manuscript and agree to be accountable for all aspects of the work in ensuring that questions related to the accuracy or integrity of any part of the work are appropriately investigated and resolved. All persons designated as authors qualify for authorship, and all those who qualify for authorship are listed.

## CONFLICT OF INTEREST

None declared.

## FUNDING INFORMATION

None.

## GENERATIVE AI STATEMENT

During manuscript, the authors used ChatGPT (OpenAI, San Francisco, CA, USA; GPT‐5.5) as a language‐support tool to assist with improving the clarity, grammar and readability of the manuscript. The AI tool was not used for study design, data collection, data analysis, data interpretation, generation of results or scientific decision‐making. All scientific content, interpretations, revisions and final manuscript text were reviewed, verified and approved by the authors, who take full responsibility for the content of the manuscript.

## Data Availability

Owing to the sensitive nature of the data, they is not publicly available. However, they can be accessed from the corresponding author upon reasonable request.
